# *LCTL* Is a Prognostic Biomarker and Correlates With Stromal and Immune Infiltration in Gliomas

**DOI:** 10.3389/fonc.2019.01083

**Published:** 2019-10-15

**Authors:** Jun Su, Qianquan Ma, Wenyong Long, Hailin Tang, Changwu Wu, Mei Luo, Xiangyu Wang, Kai Xiao, Yang Li, Qun Xiao, Chi Zhang, Haoyu Li, Qing Liu

**Affiliations:** ^1^Department of Neurosurgery, Xiangya Hospital, Central South University, Changsha, China; ^2^Department of Neurosurgery, Peking University Third Hospital, Peking University, Beijing, China; ^3^Sun Yat-sen University Cancer Center, State Key Laboratory of Oncology in South China, Collaborative Innovation Center for Cancer Medicine, Guangzhou, China; ^4^Institute of Skull Base Surgery & Neuro-oncology at Hunan, Changsha, China

**Keywords:** *Klotho*, *LCTL*, immune infiltration, methylation, FGF signaling, glioma

## Abstract

Immune evasion in glioma strongly correlates with clinical outcomes; however, the molecular mechanisms driving the maintenance of immunosuppression remain largely unknown. Recently studies demonstrate that Klothos are aberrantly expressed in several cancers and are potential therapeutic targets in cancers. However, their roles are still unclear in glioma. Here, we show that *LCTL* is highly expressed in gliomas and that its expression is regulated by DNA methylation status at the promoter. *LCTL* expression is also found to be significantly associated with high tumor aggressiveness and poor outcomes for glioma patients. Mechanistically, results suggested that *LCTL* might play an important immunosuppressive role by recruiting immunosuppressive cells and regulating tumor-associated macrophages polarization, T cell exhaustion, and epithelial–mesenchymal transition through FGF signaling in glioma. Our results establish *LCTL* as a key biomarker for prognosis that could be considered a potential epigenetic and immunotherapeutic target for treatment.

## Introduction

Gliomas are the most common primary tumors of the central nervous system and account for nearly 75% of malignant brain tumors in adults, of which glioblastoma multiform (GBM) is categorized as the most malignant subtype (1, 2). Despite current standard multimodal treatment, including maximal safe resection followed by combined radio-chemotherapy, the median survival time for patients with glioma is still <2 years (3, 4). Glioma resistant to advanced therapeutic strategies has been widely reported as a consequence of distinct metabolic mechanisms and the complicated immunosuppressive microenvironment that surrounds the tumor niche (5, 6). With advancements in molecular biology, a number of significant genetic alterations (IDH mutation, 1p/19q codeletion, H3ys27Met, and RELA-fusion) are now associated with heterogeneous tumor histology and are clinically significant based on the revised 2016 World Health Organization (WHO) classification (7). Strengthening the knowledge of such molecular alterations will ultimately contribute to a more comprehensive understanding of the diagnosis, classification, and treatment of gliomas. From this perspective, it is urgent to identify novel molecular targets and biomarkers to develop efficient therapeutic strategies.

*Klotho* was originally identified as an anti-aging gene in 1997 (8). The *Klotho* family comprises three classic members, namely *Klotho (KL)*, β*Klotho (KLB)*, and γ*Klotho* (also referred as *LCTL*) (9). All of these three genes encode transmembrane proteins, belonging to glycosidase family 1 and sharing structural similarities to β-glycosidases. However, the critical residues required for enzymatic activity are not conserved in any of the Klotho proteins (10), which indicates that their biological function is independent of glycosidase activity. Klothos proteins are known as essential cofactors that participate in interactions between fibroblast growth factors (FGFs) and fibroblast growth factor receptors (FGFRs) (9, 11). FGF and FGFRs regulate a wide range of biological functions including cell fate, angiogenesis, immunity, and metabolism, and are aberrantly activated during carcinogenesis (12, 13). In addition, recent studies have demonstrated aberrant expression of *Klothos* in several cancers including breast cancer, lung cancer, and hepatocellular carcinoma (9, 14–17). Most studies indicate that *KL* functions as a tumor suppressor and modulates several signaling pathways such as insulin-like growth factor-1 (IGF-1), FGF, and Wnt/β-Catenin (18, 19). However, the role of *KLB* in cancer is controversial. Poh et al. (17) reported that it is up-regulated and associated with FGFR4 signaling in hepatocellular carcinoma. In contrast, Ye et al. (20) reported *KLB* is down-regulated in this disease and regulates Akt/GSK-3β/cyclin D1 signaling. Although mounting evidence suggests potential connections between the *klotho* family and tumor development, the role of *LCTL* in tumorigenesis is still uncertain. Recently, Trost et al. (9) reported that *LCTL* is a potential oncogene in triple negative breast cancer and that it is necessary for resistance to increased oxidative damage. *LCTL* was also reported to be associated with cell proliferation, apoptosis, and epithelial-mesenchymal transition (EMT) in urothelial carcinoma of the bladder (21). However, the role and clinical importance of Klothos is still unclear with respect to glioma.

In this study, we first performed differential gene expression analysis of Klothos comparing the TCGA databases for glioma and normal brain tissues via a bioinformatics approach. We found of *Klotho* genes, only *LCTL* met the preset thresholds of differential expression. Further analysis revealed that the expression of *LCTL* is significantly elevated in gliomas compared to that in normal brain tissues, and that it is strictly associated with pathologic and molecular characteristics of different mutants. Moreover, by analyzing certain prognostic outcomes, *LCTL* was found to be associated with overall survival time in patients with glioma. Similar to that with KL, DNA methylation of the *LCTL* promoter can silence its endogenous expression. After identifying that high *LCTL* expression might lead to glioma progression, we further investigated its potential biological role in glioma based on gene ontology (GO) analysis and observed that it is markedly involved in tumor-associated immune responses in this disease. Further analysis revealed that increased *LCTL* expression is particularly associated with various immunosuppressive behaviors such as the recruitment of suppressive immune cells, secretion of cytokines, and transformation to tumor-promoting phenotype via FGF signaling, which in turn impairs normal immunosurveillance and leads to disease progression.

## Materials and Methods

### Data Sets and Human Tissue Samples

The Patient clinical annotation and gene expression data used in this study were obtained from publicly available databases. The TCGA lower grade glioma and glioblastoma (GBMLGG) dataset, which included genomic data and phenotypic data, was obtained from the University of California, Santa Cruz, Xena browser (https://xenabrowser.net/). The genomic data set contained DNA methylation and gene expression RNAseq (IlluminaHiSeq) data, whereas the phenotype dataset contained demographic, clinical, pathological, and IDH status. An additional data set was obtained from the Gene Expression Omnibus (GEO) GSE16011 (*n* = 284), which includes lower grade glioma and glioblastoma; and the data are also available on the R2: Genomics Analysis and Visualization Platform (http://r2.amc.nl).

In addition, we obtained glioma tissue samples from 45 patients (15 grade II, 15 grade III, and 15 grade IV glioma) and 15 normal brain tissue samples from the Department of Neurosurgery, Xiangya Hospital, Hunan, China. This study was approved by the Ethics Committee of Xiangya Hospital, Central South University and informed consent was obtained from all the patients. Tissues were frozen in RNAlater (Ambion) in liquid nitrogen and stored until total RNAs were extracted.

### Differential Expression Analysis

Gene Differential gene expression analysis was performed using the online database Gene Expression Profiling Interactive Analysis (GEPIA). GEPIA (22) is an interactive web platform for gene expression analysis, which includes 9,736 tumors and 8,587 normal samples from TCGA and GTEx databases and its gene expression data have been re-computed from raw RNA-Seq data based on the UCSC Xena project and a uniform pipeline for solving the imbalance between tumor and normal data. The preset differential thresholds were *p*-value < 0.05 and |log_2_FC| > 1. Based on differential analysis, the expression data are first log2(TPM + 1)-transformed and the log2FC is defined as median (tumor)—median (normal). Only genes that meet the preset thresholds are considered differentially expressed.

### Gene Expression Analysis

Gene expression data from TCGA were generated using the Illumina HiSeq 2000 RNA Sequencing platform, and this dataset shows gene-level transcription estimates, as log2(x + 1)-transformed RSEM normalized counts. Gene expression data from GSE16011 was generated using an Affymetrix Gene Chip Human Genome U133 Plus 2.0 Array. In addition, *LCTL* expression in diverse cancers was analyzed via Tumor Immune Estimation Resource (TIMER, http://cistrome.org/TIMER/) (23).

### Analysis of Genetic Alterations and DNA Methylation

The Genetic alterations analysis was performed using The cBioPortal for Cancer Genomics (http://cbioportal.org), which provides a web resource to explore, visualize, and analyze multidimensional cancer genomics data (24). DNA promoter methylation data (Methylation 450k), similar to gene expression data, were also downloaded from UCSC Xena browser. Pearson correlation analysis of gene expression and DNA methylation was performed and evaluated via R language.

### Survival Analysis

Kaplan–Meier survival analysis and the Cox proportional hazard model were used to estimate the prognostic value of *LCTL* based on TCGA GBMLGG data using R language packages (survival and survminer). Kaplan–Meier survival analysis for the GSE16011 data set was generated by the R2: Genomics Analysis and Visualization Platform.

### Gene Ontology (GO) Enrichment Analysis

Gene ontology enrichment analysis was performed by The Database for Annotation, Visualization and Integrated Discovery (DAVID, https://david.ncifcrf.gov/), an online software, to identify GO categories by their biological processes, molecular functions, and cellular components (25). Enriched ontological terms with *P* < 0.05 were regarded as statistical significance.

### Analysis of Stromal and Immune Infiltration

ESTIMATE (Estimation of STromal and Immune cells in MAlignant Tumor tissues using Expression) algorithm was described by Yoshihara (26) to assess the presence of stromal cells and the infiltration of immune cells in tumor samples, and its predictive ability has been validated in large and independent data sets. The ESTIMATE algorithm generates three scores as follows: stromal score (positively correlating with the presence of stroma in tumor tissue), immune score (positively correlating with the level of immune cells infiltrations in tumor tissue), and estimate score (which infers and negatively correlates with tumor purity). xCell, reported by Aran (27) to estimate the enrichment of cell types, can estimate 64 cell types, spanning multiple adaptive and innate immune cells, hematopoietic progenitors, epithelial cells, and extracellular matrix cells derived from expression profiles. The scores, calculated by the ESTIMATE algorithm, were downloaded from https://bioinformatics.mdanderson.org/estimate/. The pre-calculated TCGA data based on xCell was downloaded from http://xcell.ucsf.edu/. Then the correlation between *LCTL* expression and ESTIMATE scores and 64 cell types from the TCGA glioma dataset were analyzed using R language.

### Protein-Protein Interaction (PPI) Analysis

The Search Tool for the Retrieval of Interacting Genes (STRING), an online database, was used to identify proteins that can interact with *LCTL* and construct PPI networks for this protein, immunosuppressive cell recruitment factors, immunosuppressive factors, factors promoting M2 differentiation, and markers of T cell exhaustion and EMT.

### Cell Lines and Culture

The human glioma cell lines (U87, U251, SF126, SF767, A172, and SHG-44) and the normal glial cell line HEB were obtained from Sun Yat-Sen University Cancer Center. All the cell lines were cultured in DMEM with 10% FBS and antibiotics (100 μg/ml penicillin and 100 μg/ml streptomycin), and maintained in standard culture condition.

### RNA Extraction and Real-Time PCR

Total RNA was extracted from cell lines or human tissues by Trizol reagent (Invitrogen) according to the manufacturer's protocol. Then, total RNA was quantified and 1 μg of RNA was reverse-transcribed with the Reverse Transcription Kit (Thermo Fisher Scientific). Q-PCR was performed using SYBR Premix Ex Taq II (Takara Bio). β-Actin mRNA was used to normalize the expression of genes. Primers are described in Supplementary Table S1.

### Statistical Analysis

Statistical computations and the creation of figures were performed with several packages (ggplot2, survival, survminer, corrplot) in the statistical software environment R, version 3.5.3 (http://www.r-project.org).

## Results

### *LCTL* mRNA Expression Levels in Gliomas and Other Cancers

**Figure 1:**
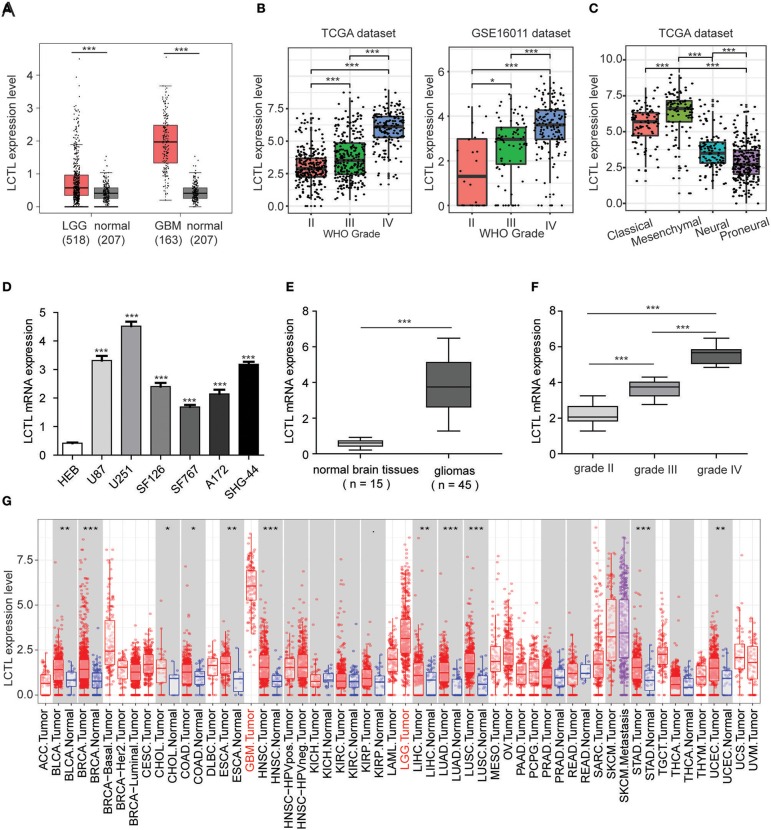
*LCTL* is highly expressed in gliomas and significantly associated with tumor aggressiveness. **(A)** Differential expression of *LCTL* in brain lower grade glioma (LGG) and glioblastoma (GBM) compared to levels in normal brain tissues. **(B)** *LCTL* expression in glioma of WHO grade II–IV based on both TCGA and GSE16011 datasets. **(C)** *LCTL* expression pattern in different molecular subtypes of glioma (classical, mesenchymal, neural, proneural) in the TCGA dataset. **(D)** The mRNA expression level of *LCTL* in glioma cell lines and the normal glial cell line HEB by Q-PCR. **(E)** Validation of aberrant mRNA expression of *LCTL* in 45 gliomas compared to 15 normal brain tissues via Q-PCR. **(F)** *LCTL* expression in glioma of WHO grade II–IV based on our patient samples. **(G)** Expression of *LCTL* in multiple cancers as determined by TIMER analysis. ACC, Adrenocortical carcinoma; BLCA, Bladder Urothelial Carcinoma; BRCA, Breast invasive carcinoma; CESC, Cervical squamous cell carcinoma and endocervical adenocarcinoma; CHOL, Cholangiocarcinoma; COAD, Colon adenocarcinoma; DLBC, Lymphoid Neoplasm Diffuse Large B-cell Lymphoma; ESCA, Esophageal carcinoma; GBM, Glioblastoma multiforme; HNSC, Head and Neck squamous cell carcinoma; KICH, Kidney Chromophobe; KIRC, Kidney renal clear cell carcinoma; KIRP, Kidney renal papillary cell carcinoma; LAML, Acute Myeloid Leukemia; LGG, Brain Lower Grade Glioma; LIHC, Liver hepatocellular carcinoma; LUAD, Lung adenocarcinoma; LUSC, Lung squamous cell carcinoma; MESO, Mesothelioma; OV, Ovarian serous cystadenocarcinoma; PAAD, Pancreatic adenocarcinoma; PCPG, Pheochromocytoma and Paraganglioma; PRAD, Prostate adenocarcinoma; READ, Rectum adenocarcinoma; SARC, Sarcoma; SKCM, Skin Cutaneous Melanoma; STAD, Stomach adenocarcinoma; TGCT, Testicular Germ Cell Tumors; THCA, Thyroid carcinoma; THYM, Thymoma; UCEC, Uterine Corpus Endometrial Carcinoma; UCS, Uterine Carcinosarcoma; UVM, Uveal Melanoma. **P < 0.01, ***P < 0.001.

To assess the differential expression of *Klotho* genes in gliomas and normal brain tissues, the online database GEPIA was used. Results showed that there was no significant difference in the expression of *KL* between gliomas and normal brain tissues (*p* > 0.05, Supplementary Figure S1A). Even though the expression of *KLB* was significantly downregulated in glioma compared to levels in normal brain tissue (*p* < 0.05, Supplementary Figure S1B), it was still not a differentially expressed, as the |log_2_FC| value was <1. Interestingly, among *Klotho* genes, only *LCTL* met the preset criterion and was significantly upregulated in both brain lower grade glioma (LGG) and GBM, as compared to levels in normal tissue (*p* < 0.05, Figure 1A). We further analyzed the expression of this gene in different grades of glioma and found that its level significantly increases with WHO grades based on the TCGA dataset (Figure 1B, left). Further, this result was validated by the GSE16011 dataset (Figure 1B, right). In addition, *LCTL* expression was significantly upregulated in the mesenchymal subtype compared with other three respective molecular subtypes in the TCGA dataset (Figure 1C). To further validate these findings, Q-PCR was performed in cell lines and our 45 gliomas samples. The results showed that *LCTL* was upregulated in glioma cell lines compared with that in normal glial cell line (Figure 1D), its expression level was higher in glioma tissues than that in normal brain tissues (Figure 1E), and the expression level of *LCTL* positively correlated with WHO grade of gliomas (Figure 1F). Moreover, an analysis of *LCTL* expression in multiple human cancer types via TIMER showed that the expression levels were highest in GBM, following by skin cutaneous melanoma (SKCM) and LGG (Figure 1G). These results indicated that *LCTL* is significantly up regulated in glioma and several other cancers.

### *LCTL* Expression Is Associated With Glioma Patient Outcomes

**Figure 2:**
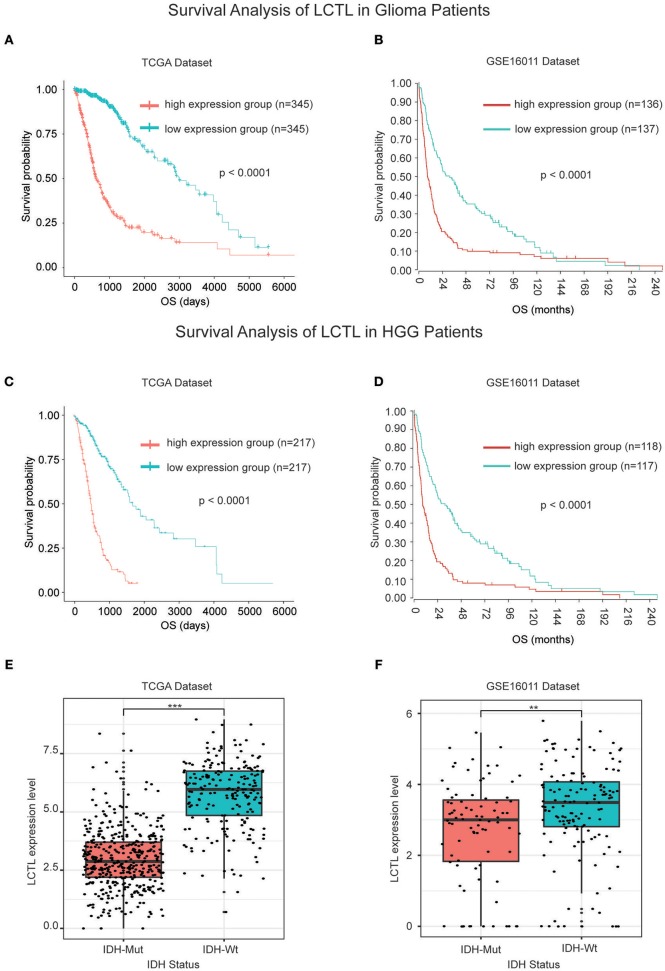
*LCTL* is a prognostic factor for glioma patients. **(A,B)** Kaplan–Meier survival analysis showing that high *LCTL* expression predicts poor prognosis for glioma patients based on both the TCGA and GSE16011 datasets. **(C,D)** Kaplan–Meier survival analysis showing that high *LCTL* expression predicts poor prognosis for high grade glioma (HGG) patients in both the TCGA and GSE16011 datasets. **(E,F)** The expression of *LCTL* is significantly higher in IDH-Wt gliomas than that in IDH-Mut disease based on both the TCGA and GSE16011 datasets. **P < 0.01, ***P < 0.001, t-test.

Since the expression of *LCTL* was found to be aberrantly expressed in gliomas and to correlate with histological grade and molecular subtype, we further studied its prognostic value. For this, Kaplan–Meier survival analysis was performed to evaluate the predictive effects of this gene using both TCGA and GSE16011 datasets. Results indicated that high *LCTL* expression is not only significantly associated with poor prognosis in glioma patients (Figures 2A,B), but also in patients with high grade glioma (Figures 2C,D). Recently, increasing evidence has suggested recurrent point mutations in isocitrate dehydrogenase genes (*IDH1* and *IDH2*) occur in specific types of glioma (28). Although tumors exhibit identical histologies, the outcome for IDH-mutant (IDH-Mut) diffuse gliomas is better than that with IDH-wildtype (IDH-Wt) disease (29). Further, in the latest version of the WHO Classification of Central Nervous System Tumors published in 2016, IDH mutations were adopted as a decisive marker for glioma classification (30). Currently, this is widely used clinically as a strong prognostic marker for glioma patients. Interestingly, we also found that *LCTL* expression in IDH-Wt gliomas was significantly higher than that in IDH-Mut tumors based on TCGA dataset (Figure 2E), consistent with results obtained from the GSE16011 dataset (Figure 2F). Simultaneously, we wondered whether *LCTL* could be an independent prognostic marker for glioma and performed univariate and multivariate Cox regression analysis based on the TCGA dataset. Univariate analysis revealed that *LCTL* expression, patient age at diagnosis, WHO grade, and IDH status, were significantly associated with overall survival. Based on multivariate analysis, the expression of *LCTL* was also a significant predictive factor after adjusting for the aforementioned clinical factors (Table 1). Taken together, *LCTL* is an independent prognostic factor, and high expression indicates poor clinical outcome for glioma patients.

**Table 1:** Univariate and multivariate analysis of clinical prognostic parameters based on the TCGA Dataset.

	**Univariate analysis**			**Multivariate analysis**		
**Variable**	**HR**	**95% CI**	***p***	**HR**	**95% CI**	***p***
*LCTL*	1.685	1.562–1.816	<0.001	1.167	1.051–1.296	<0.01
Age	1.072	1.061–1.083	<0.001	1.035	1.022–1.047	<0.001
WHO grade	10.670	7.815–14.56	<0.001	1.983	1.352–2.907	<0.001
IDH status	9.991	7.425–13.440	<0.001	3.577	2.317–5.523	<0.001
Gender	1.113	0.848–1.461	0.4	1.228	0.930–1.619	0.147

### Promoter DNA Methylation Regulates *LCTL* mRNA Expression

**Figure 3:**
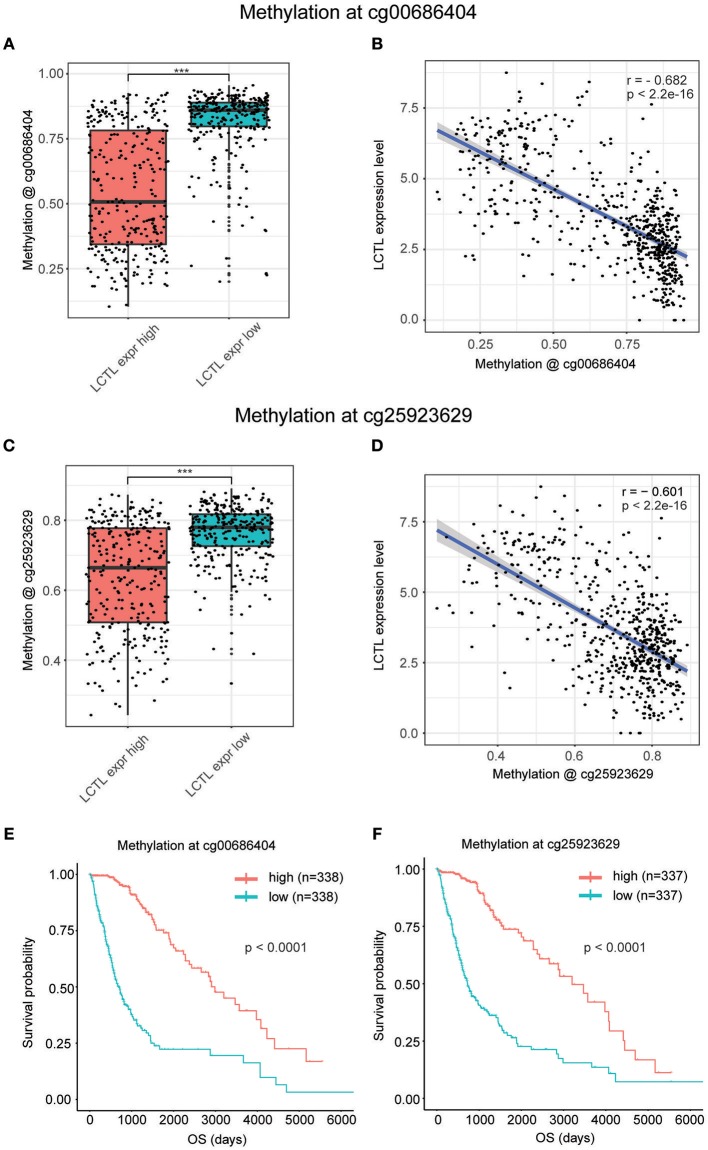
Promoter DNA methylation regulates *LCTL* mRNA expression and correlates with glioma patient prognosis. **(A)** Comparison of DNA methylation beta values at cg0686404. **(B)** Correlation between *LCTL* mRNA expression and promoter DNA methylation at the probe cg00686404. **(C)** Comparison of DNA methylation beta values at cg25923629. **(D)** Correlation between *LCTL* mRNA expression and promoter DNA methylation at the probe cg25923629. **(E)** Association between the promoter DNA methylation value of *LCTL* at cg0686404 overall survival time for glioma patients. **(F)** Association between the promoter DNA methylation value of *LCTL* at cg25923629 and overall survival time for glioma patients. ***P < 0.001, t-test.

It is widely recognized that both genetic alterations (mutations, loss of heterozygosity, deletions, insertions, aneuploidy, etc.) and epigenetic alterations (DNA methylation, non-coding RNAs, transcription factors, etc.) equally contribute to carcinogenesis (31). Whereas, the former perturb normal patterns of gene expression, which is dependent on changes to normal DNA sequences, the latter result in the inappropriate silencing or activation of cancer-associated genes without changing DNA sequences (32). Interestingly, after the analysis of genetic alterations by cBioPortal, we found no genetic alterations including mutations and putative copy-number alterations in *LCTL* in TCGA Merged Cohort of LGG and GBM (Supplementary Table S2). Next, we investigated epigenetic alterations of *LCTL*, and especially promoter DNA methylation. After integrating TCGA glioma datasets, we identified 593 patients with both *LCTL* expression and DNA methylation data. The *LCTL* DNA methylation beta values at cg00686404 were significantly higher in the low *LCTL* expression group than in the high *LCTL* expression group (Figure 3A). Meanwhile, the beta values at cg00686404 were negatively correlated with *LCTL* mRNA expression (Pearson's *r* = −0.682, *p* < 2.2e-16; Figure 3B). Consistently, an analysis of DNA methylation at cg25923629 generated similar results (Figures 3C,D). To further validate our previous results, we next analyzed the predictive value of *LCTL* promoter DNA methylation for glioma patients and found that patients with high levels had significantly better prognosis than those with low levels (Figures 3E,F, *p* < 0.0001). In summary, these results indicate that the expression of *LCTL* is likely regulated by DNA methylation of its promoter, and that this epigenetic modification might also represent a potential prognostic marker for glioma patients.

### *LCTL* Related GO Functional Enrichment in Glioma

**Figure 4:**
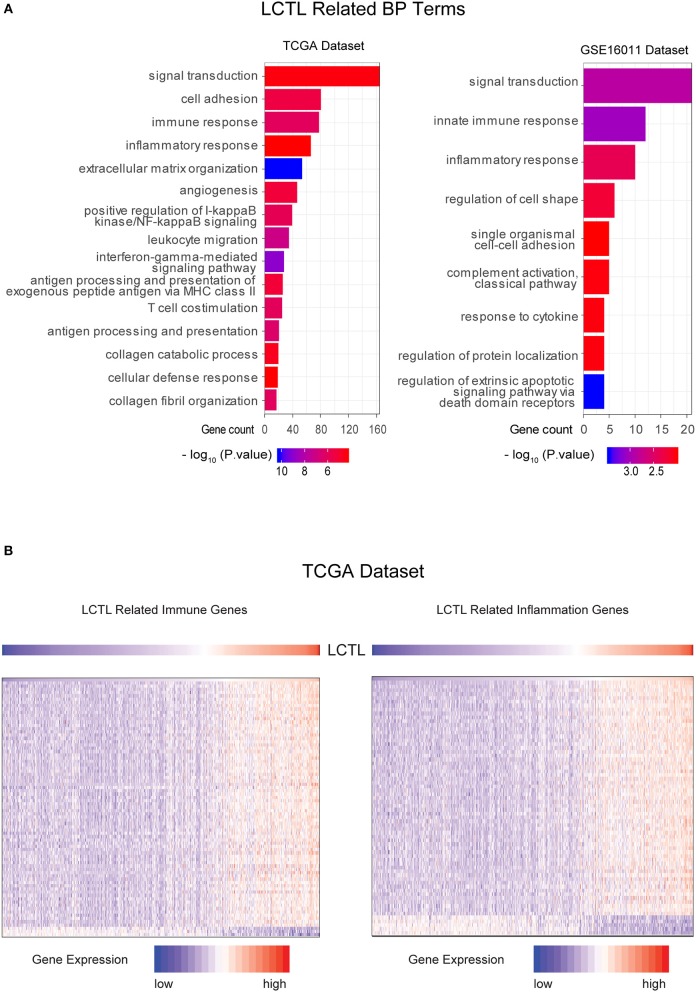
*LCTL* is related to specific gene ontology (GO) terms in glioma. **(A)** Results of BF (biological function) terms analyzed by DAVID based on the TCGA and GSE16011datasets. **(B)** Most immune response and inflammatory response related genes were significantly positively correlated with *LCTL*.

To further clarify the biologic role of *LCTL* in glioma, GO enrichment analysis was performed. First, we analyzed the correlation between *LCTL* and all other genes in the TCGA dataset by Pearson correlation analysis and in the GSE16011 dataset by R2. Genes with |R| > 0.5 in both data sets were chosen for further analysis. Finally, we obtained 2091 genes and 161 genes from TCGA and GSE16011 gene lists, respectively (Supplementary Table S3). Then, we probed the bio-function of these genes by GO analysis in DAVID Bioinformatics Resources 6.8 (Supplementary Tables S4, S5) and found that genes that were tightly correlated with *LCTL* expression in TCGA and GSE16011 datasets were enriched in 66 biological processes terms (the top 15 terms are shown in Figure 4A left, *p*-value < 0.01) and nine terms (Figure 4A right, *p*-value < 0.01), respectively. When comparing the two lists of GO terms, we found that genes closely related with *LCTL* were involved in the immune response and inflammatory response for both TCGA and GSE16011 datasets. Regarding molecular function, these genes were mainly enriched in protein binding (Supplementary Figures S1C,D). Moreover, cell component analysis indicated enrichment predominantly occurred at the extracellular region (Supplementary Figures S1E,F), which indicated that these genes might play a vital role in the tumor microenvironment (TME) of glioma. Importantly, the majority of genes that were enriched in immune response and inflammatory response in the TCGA dataset were significantly positively correlated with *LCTL* expression (Figure 4B and Supplementary Figure S2). Collectively, these findings suggest that *LCTL* participates in regulating the tumor immune environment.

### *LCTL* Expression Is Correlated With Stromal and Immune Cell Infiltration in Gliomas

**Figure 5:**
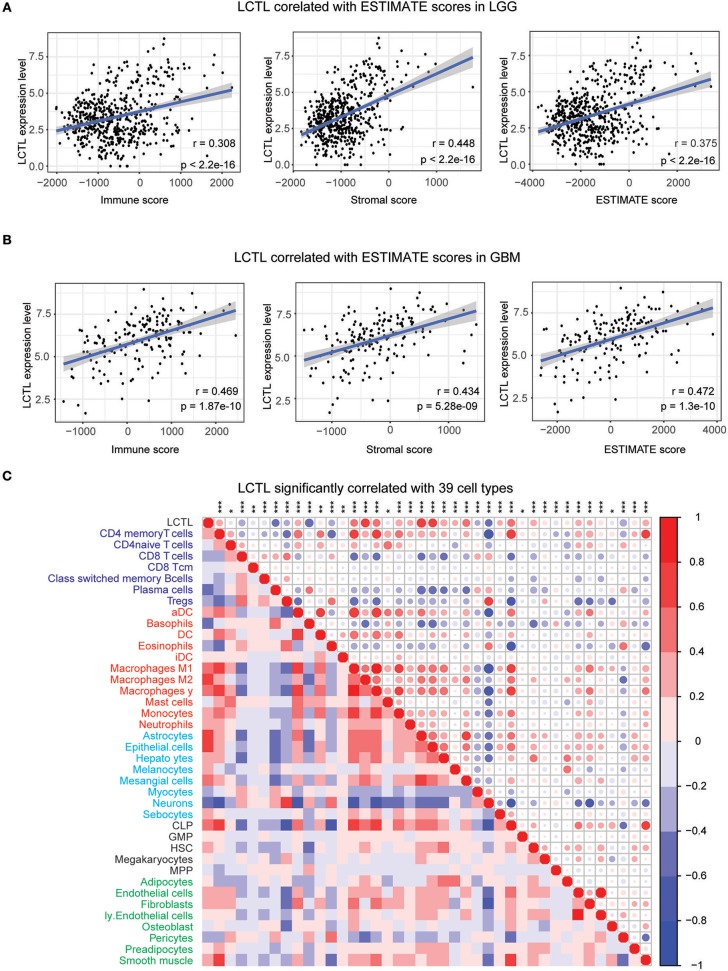
Correlation between *LCTL* expression and ESTIMATE algorithm/xcells scores in glioma. **(A)** *LCTL* expression was positively correlated with immune score, stromal score and ESTIMATE score in lower grade glioma (LGG) patients. **(B)** *LCTL* expression was positively correlated with immune score, stromal score and ESTIMATE score in glioblastoma (GBM) patients. **(C)** *LCTL* expression was significantly correlated with 39 cell types, as calculated by xcells in glioma. *P < 0.05, **P <0.01, ***P < 0.001.

Infiltrating stromal and immunes cells, which form the major normal cells component of tumors, play an important role in cancer biology and perturb tumor signaling based on molecular studies (26). Considering the results of GO analysis, we next explored whether *LCTL* expression is associated with immune infiltration in glioma. First, we examined the association between *LCTL* expression and ESTIMATE scores. Results revealed that *LCTL* expression was significantly positively correlated with stromal score, immune score, and ESTIMATE score in both LGG (Figure 5A) and GBM patients (Figure 5B), suggesting that it has a marked influence on stromal and immune cell infiltration. Since the immune and stromal scores were, respectively, generated based on their gene signatures (26), we therefor performed Q-PCR to investigate the association between mRNA expression of *LCTL* and typical genes of immune and stromal scores in our 45 glioma samples. The results demonstrated that mRNA expression of *LCTL* significantly correlated with that of selected typical genes (top genes correlated with immune and stromal scores in TCGA GBMLGG cohort, Supplementary Figure S3). To further determine which cell types play a predominant role in this process, we next analyzed the correlation between *LCTL* expression and 64 non-cancerous cell types, as estimated by xcells, based on TCGA glioma patients. The results showed that there were 39 cell types that significantly correlated with *LCTL* expression (Figure 5C and Table 2, Spearman's *r*, BH-adjust *p*-value < 0.05), among which, 25 types were positively correlated, whereas 14 types were negatively correlated. These cells types comprised seven lymphoid, 11 myeloid cells, eight stromal, five stem, and eight other cell types. Notably, the majority of myeloid cells, stromal cells, stem cells, and other cells were positively correlated with *LCTL* expression; however, most lymphoid cells were negatively correlated. These findings strongly indicate that *LCTL* plays a specific role in stromal and immune cell infiltration in gliomas.

**Table 2:** Correlation ship between *LCTL* and 64 types of non-cancerous cells.

**xCells**	**Category**	***r* value (Spearman)**	**CI adjust**	**Ajust. *P*. val (BH)**
Plasma cells	Lymphoids	−0.453	−0.573~−0.312	2.69E-33
CD8+T cells	Lymphoids	−0.346	−0.482~−0.193	5.81E-19
CD4+ memory T cells	Lymphoids	0.336	0.183~0.473	6.99E-18
Tregs	Lymphoids	−0.279	−0.423~−0.121	1.89E-12
Class switched memory B cells	Lymphoids	−0.18	−0.333~−0.017	1.28E-05
CD8+ Tcm	Lymphoids	−0.128	−0.285~0.036	2.90E-03
CD4+ naive T cells	Lymphoids	0.093	−0.252~0.072	3.91E-02
Th2 cells	Lymphoids	0.071	−0.093~0.232	1.30E-01
NK cells	Lymphoids	−0.059	−0.220~0.105	2.25E-01
CD4+ Tcm	Lymphoids	−0.059	−0.220~0.106	2.31E-01
CD8+ Tem	Lymphoids	0.056	−0.108~0.217	2.59E-01
CD8+ naive T cells	Lymphoids	0.041	−0.123~0.203	4.39E-01
B cells	Lymphoids	0.034	−0.130~0.196	5.36E-01
naive B cells	Lymphoids	−0.031	−0.193~0.133	5.73E-01
Th1 cells	Lymphoids	−0.031	−0.193~0.133	5.80E-01
Memory B cells	Lymphoids	0.016	−0.147~0.179	7.87E-01
CD4+ T cells	Lymphoids	−0.015	−0.178~0.148	8.01E-01
Tgd cells	Lymphoids	0.010	−0.154~0.173	8.84E-01
CD4+Tem	Lymphoids	−0.007	−0.170~0.156	9.15E-01
pro B cells	Lymphoids	−0.006	−0.169~0.157	9.29E-01
Natural killer T cells (NKT)	Lymphoids	0.000	−0.163~0.163	9.97E-01
Macrophages M2	Myeloids	0.618	0.506~0.710	5.99E-69
Basophils	Myeloids	−0.559	−0.662~−0.436	6.81E-54
Macrophages y	Myeloids	0.460	0.320~0.579	1.87E-34
Macrophages M1	Myeloids	0.436	0.294~0.559	1.04E-30
Monocytes	Myeloids	0.365	0.215~0.499	3.64E-21
Eosinophils	Myeloids	−0.321	−0.460~−0.167	2.17E-16
Activated dendritic cells (aDC)	Myeloids	0.312	0.157~0.452	1.81E-15
Neutrophils	Myeloids	0.256	0.096~0.402	1.51E-10
Immature DC (iDC)	Myeloids	0.126	−0.037~0.284	3.23E-03
Denritic cells (DC)	Myeloids	0.125	−0.039~0.282	3.67E-03
Mast cells	Myeloids	−0.092	−0.251~0.072	4.11E-02
Xonventional dendritic cells (cDC)	Myeloids	−0.035	−0.197~0.129	5.24E-01
Plasmacytoid dendritic cells (pDC)	Myeloids	−0.025	−0.188~0.138	6.59E-01
Astrocytes	Others	0.681	0.583~0.760	3.58E-89
Epithelial cells	Others	0.601	0.486~0.696	1.89E-64
Mesangial cells	Others	0.464	0.326~0.583	3.22E-35
Neurons	Others	−0.420	−0.546~−0.276	2.18E-28
Hepatocytes	Others	0.361	0.210~0.495	1.25E-20
Melanocytes	Others	0.257	0.098~0.404	1.09E-10
Myocytes	Others	−0.226	−0.375~−0.065	2.14E-08
Sebocytes	Others	0.222	0.061~0.372	3.91E-08
Keratinocytes	Others	0.031	−0.133~0.193	5.79E-01
Common lymphoid progenitors (CLP)	Stem cells	0.466	0.327~0.584	1.86E-35
Hematopoietic stem cells (HSC)	Stem cells	0.317	0.162~0.457	6.06E-16
Multipotent rogenitors (MPP)	Stem cells	−0.169	−0.323~−0.006	4.72E-05
Megakaryocytes	Stem cells	0.159	−0.005~0.314	1.42E-04
Granulocyte-macrophage progenitorGMP	Stem cells	0.100	−0.065~0.259	2.50E-02
Platelets	Stem cells	−0.030	−0.192~0.134	5.91E-01
Common myeloid progenitors (CMP)	Stem cells	0.021	−0.142~0.184	7.15E-01
Megakaryocyte–erythroid progenitors (MEP)	Stem cells	0.009	−0.154~0.172	8.96E-01
Erythrocytes	Stem cells	−0.005	−0.168~0.159	9.48E-01
Pericytes	Stromal cells	−0.396	−0.525~−0.249	4.61E-25
Smooth muscle	Stromal cells	0.316	0.161~0.456	6.93E-16
Endothelial cells	Stromal cells	0.290	0.133~0.433	2.20E-13
Fibroblasts	Stromal cells	0.213	0.051~0.363	1.56E-07
Preadipocytes	Stromal cells	0.199	0.037~0.351	1.06E-06
ly Endothelial cells	Stromal cells	0.193	0.031~0.345	2.39E-06
Adipocytes	Stromal cells	0.142	−0.022~0.298	8.01E-04
Osteoblast	Stromal cells	−0.110	−0.269~0.054	1.18E-02
mv Endothelial cells	Stromal cells	0.080	−0.084~0.240	8.21E-02
Skeletal muscle	Stromal cells	−0.046	−0.208~0.118	3.66E-01
Mesenchymal stem cells (MSC)	Stromal cells	−0.044	−0.206~0.120	3.98E-01
Chondrocytes	Stromal cells	0.032	−0.132~0.194	5.67E-01

### *LCTL* Correlates With Immunosuppressive Properties

**Figure 6:**
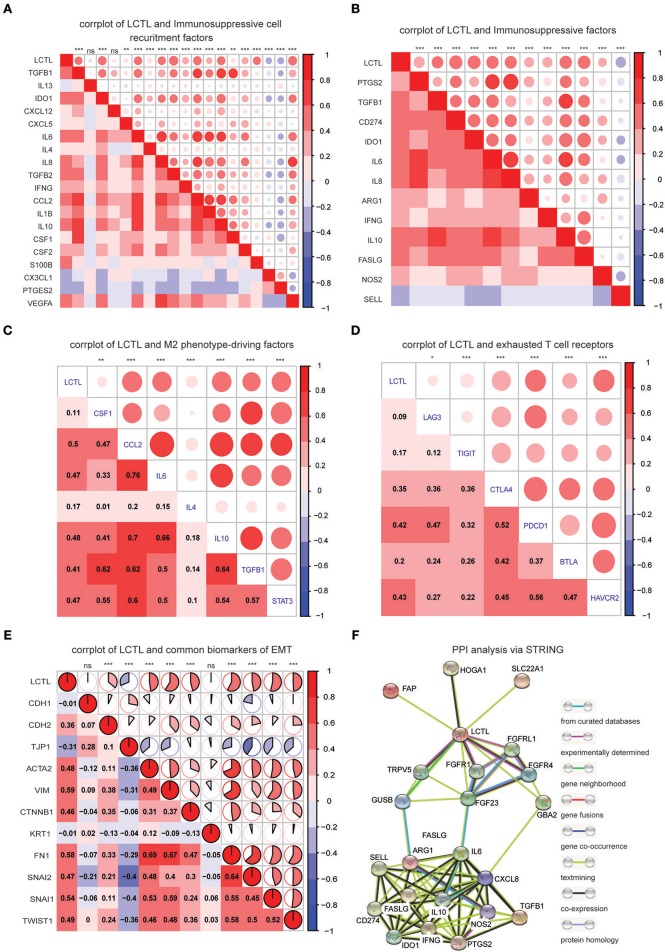
*LCTL* correlates with immunosuppressive properties and promotes epithelial-mesenchymal transition (EMT). **(A)** Correlation between *LCTL* and immunosuppressive cell recruitment factors. **(B)** Correlation between *LCTL* and immunosuppressive factors secreted by myeloid-derived suppressor cells (MDSCs), tumor-associated macrophages (TAMs) and tumor-associated neutrophils (TANs). **(C)** Correlation between *LCTL* and M2 phenotype-driving factors. **(D)** Correlation between *LCTL* and exhausted T cell receptors. **(E)** Correlation between *LCTL* and common biomarkers of epichelial-mesenchymal transition (EMT). **(F)** Protein-protein interaction (PPI) analysis of the network linking *LCTL* and immunosuppressive factors. *P < 0.05, **P <0.01, ***P < 0.001, ^*ns*^*P* > 0.05.

Since *LCTL* was found to be positively related to immunosuppressive cells, such as M2 macrophages and neutrophils, among others, but was negatively correlated with anti-tumor immune cells such as CD8+ T cells, we hypothesized that this gene could be involved in the immunosuppressive properties of glioma. To confirm this, we performed correlation analysis of *LCTL* expression and critical factors that recruit myeloid-derived suppressor cells, tumor-associated macrophages (TAMs), and tumor-associated neutrophils, as well as the immunosuppressive factors secreted by these cells (Figures 6A,B). *LCTL* was found to be significantly positively correlated with the majority of immunosuppressive cell recruitment factors [reviewed in (5)] and immunosuppressive factors [reviewed in (5, 33)]. TAMs can be divided into the “classically activated” M1 phenotype and the “alternatively activated” M2 phenotype (34). It is the M2 phenotype of TAMs that contributes to the immunosuppressive tumor environment (35). Indeed, key factors [CSF-1,CCL2, IL-4, IL-6, IL-10, TGFβ, reviewed in (5)] that drive M2 phenotype differentiation were found to be closely related to *LCTL* expression (Figure 6C). It is well-established that T cell exhaustion occurs in humans with cancer and that exhausted T cells in the TME lead to cancer immune evasion (36). Exhausted T cells express high levels of inhibitory receptors including PD-1, CTLA-4, TIM-3, LAG-3, BTLA, and TIGIT [reviewed in (36)]. To further substantiate these findings, we next analyzed the correlation between *LCTL* and inhibitory receptors of exhausted T cells (Figure 6D). Our GO enrichment analysis suggested that *LCTL* might be involved in TME, from which various pro-invasion signals can control EMT (37). Further, *LCTL* can promote EMT, as recently reported for bladder cancer (21). Consistently, we found significant correlations between *LCTL* and common EMT biomarkers, except CDH1 and KRT1 (Figure 6E). Specifically, *LCTL* expression was positively related to mesenchymal cell markers and negatively correlated with epithelial cell markers. Furthermore, we selected 14 genes, which showed the most correlation with *LCTL* in the TCGA dataset, from abovementioned immunosuppressive factors to validate the correlation between *LCTL* and immunosuppressive properties in our 45 glioma samples by Q-PCR. The results show that *LCTL* expression significantly correlated with the expression of these 14 genes at mRNA level (Supplementary Figure S4). Importantly, we found that *LCTL* can directly interact with several proteins, especially FGFRs and FGFs, via PPI analysis (Supplementary Figure S5A). Interestingly, via associations with FGFs and FGFRs, *LCTL* could indirectly interact with immunosuppressive factors, recruitment factors, M2 phenotype-driving factors, and markers of exhausted T cells and EMT (Figure 6F and Supplementary Figure S5B). Therefore, our study identifies *LCTL* as a regular of the TME in glioma, which functions by recruiting and promoting immunosuppressive cells to secrete immunosuppressive factors, in addition to regulating M2 transformation, T cell exhaustion, and EMT via the FGF signaling pathway; this is thought to ultimately facilitate tumor immune evasion, resulting in poor outcomes for glioma patients.

## Discussion

Glioma patient prognosis remains poor, which is clinically frustrating (3, 4). Even with intensive therapies, most GBM patients relapse very soon due to the highly aggressive nature of this disease. Recently, immunotherapy has shown promise for the treatment of many cancers including glioma. However, the widespread application of immunotherapy is difficult, which is attributed to autoimmune-like side effects (38, 39). One potential therapeutic strategy is to develop immunotherapy against glioma by targeting overexpressed proteins that play an essential role in immunosuppression. Our present study first identified a novel function for *LCTL* in promoting aggressive behavior and leading to immune evasion.

Originally, *Klotho*, the first identified member of the *Klotho* family, was discovered as an anti-aging gene in 1997 (8). Recently, both *Klotho* and β*Klotho* have been reported to be aberrantly expressed in several cancers (40). Although some studies have demonstrated the abnormal expression of *LCTL* in different cancers, its function was previously unclear. In this study, we found that the expression of *LCTL* is not only abnormally upregulated, but also significantly associated with WHO grade, molecular subtype, and IDH status in glioma. Furthermore, our study showed that *LCTL* is an independent prognostic marker for glioma patients and that high levels of *LCTL* expression predict poor outcome. DNA methylation, the main epigenetic modification, is also involved in the pathogenesis of cancer (41, 42). Accordingly, the methylation of promoters of certain genes involved in key biological pathways in glioma has been widely reported (43). For example, promoter methylation of the O6-methylguanine-DNA methyltransferase (*MGMT*) gene occurs in ~40% of glioma patients and is clinically used as a biomarker of response to alkylating agents (44). Because DNA methylation is potentially reversible, it is a potential target for cancer treatment. Actually, the Food and Drug Administration (FDA) has approved DNA methylation inhibitors and histone deacetylase inhibitors for cancer monotherapy (45). Here, we found that the endogenous expression of *LCTL* is regulated by promoter DNA methylation rather than genetic alterations in glioma. Moreover, *LCTL* promoter methylation levels were found to have predictive value for glioma patients, which is similar to results found for mRNA levels. Taken together, *LCTL* is a good candidate prognostic and therapeutic target for glioma patient.

Immunotherapy is rapidly becoming the newest and the most promising pillar of malignancy treatment, with the potential to harness the potency of and active the host immune system. However, the TME including stromal cells, inflammatory cells, vasculature, and ECM, usually prevents effective lymphocyte initiation, reduces their infiltration, and suppresses infiltrating effector cells, which contributes to host failure in rejecting tumors (46). It is thus of great importance to explore novel molecular biomarkers and targets that play critical roles in the TME. Here, we conducted GO analysis with two different large datasets, and the results strongly suggested that *LCTL* is involved in the regimenting immunity and the TME in glioma. Furthermore, we found that *LCTL* expression significantly correlates with non-cancerous cells, mainly stromal and immune cells, in glioma via two methods, namely the ESTIMATE algorithm and xcell. Interestingly, these infiltratimg cells such as M2 macrophages and neutrophils, among others, are mainly considered immunosuppressive cells based on previous studies. Moreover, the correlation between *LCTL* expression and marker genes encoding immunoregulatory factors, as well as exhausted T cells, imply a role for *LCTL* in regulating tumor immunology in glioma. On the one hand, *LCTL* expression was significantly positively correlated with cytokines or chemokines that recruit immunosuppressive cells, such as myeloid-derived suppressor cells, TAMs, and tumor-associated neutrophils, as well as immunosuppressive factors secreted by these cells. On the other hand, *LCTL* was found to be positively associated with factors that drive M2 phenotype differentiation, which suggests a potential regulatory role for *LCTL* in TAMs polarization. In addition, our results indicate that *LCTL* can induce T cell exhaustion. Recently, accumulating evidence has demonstrated that cross-talk between EMT-associated factors and the TME might facilitate tumor immune escape (47). Accordingly, *LCTL* was found to promote bladder cancer EMT (21). In glioma, we also found that *LCTL* expression is positively related to mesenchymal cell markers and negatively correlated with epithelial cell markers. Thus, we propose that this gene might be involved in regulation of the immunosuppressive microenvironment by recruiting and promoting immunosuppressive cells to secrete immunosuppressive factors, regulating M2 polarization, promoting EMT, and inducing T cell exhaustion in glioma, finally facilitating tumor immune evasion.

Mechanistically, *LCTL* might regulate the immunity in glioma through FGF signaling pathway. FGFR dysfunction is widely found in cancers and FGF biology is involved in many effects in a myriad of cell types, and is a key component of the tumor environment (48). In addition, increasing evidence suggests that FGF signaling can influence the recruitment and activity of TAMs. For example, the activation of inducible FGFR1 in mammary cells causes their transformation and induces the secretion of factors to recruit TAMs (49, 50). Furthermore, FGF2 secreted from esophageal cancer cells promotes macrophage migration and survival through FGFR1 signaling (51). Moreover, by cooperating with proinflammatory cytokines, FGF2 can also promote the expression of adhesion molecules by endothelial cells, thus contributing to the recruitment of circulating immune cells to the tumor (52). Indeed, a recent study demonstrated that *Klotho* regulates immune invasion in the cenral nervous system and inhibits thioredoxin-interacting protein-dependent activation of the NLRP3 inflammasome in macrophages via FGF23 signaling (53). It has also been determined that *LCTL* can interact efficiently with FGFR1b, FGFR-1c, FGFR2c, and FGFR-4 (11). In our study, we found that *LCTL* can interact with FGFR1, FGFRL1, FGFR4, and FGF23 through PPI analysis. Interestingly, this analysis also revealed that *LCTL* plays an immunoregulatory role that is dependent on FGF signaling.

**Figure 7:**
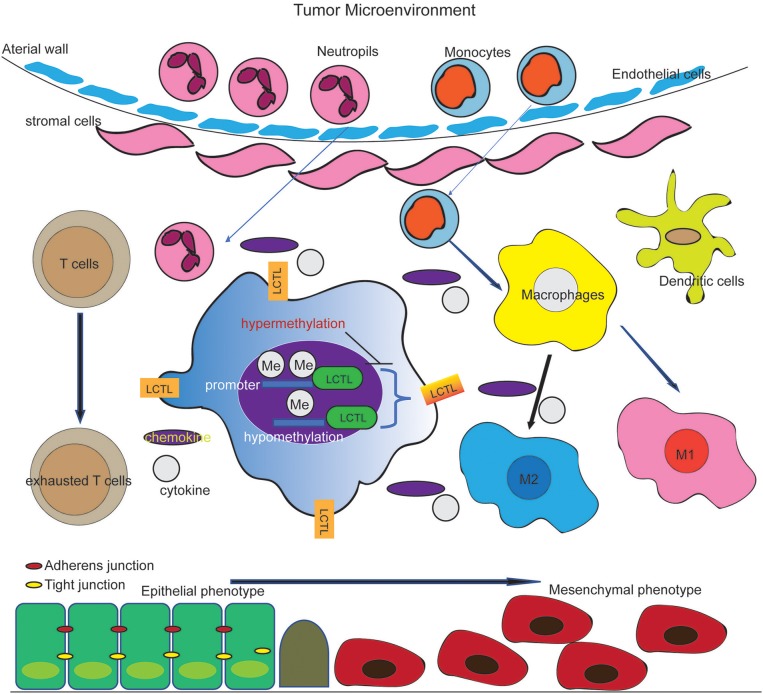
Working model of the effect of *LCTL* in the glioma microenvironment. *LCTL* is highly expressed in glioma, regulated by DNA promotor methylation and acts an immunosuppressive role via interacting with some immunosuppressive factors in glioma TME.

Based on our findings, we propose a working modal wherein *LCTL*, which is highly expressed in glioma and regulated by DNA methylation at its promoter, facilitates the recruitment of and immunosuppressive cells and promote the secretion of immunosuppressive factors by these cells. This further regulates M2 transformation, T cell exhaustion in glioma, and EMT, finally augmenting tumor immune evasion (Figure 7). Our findings provide opportunities to explore novel therapeutic approaches based on the epigenetic targeting of *LCTL*. Further, this gene might be an ideal target for immunotherapy against gliomas.

## Data Availability Statement

Publicly available datasets were analyzed in this study. This data can be found here: https://xenabrowser.net/datapages/?cohort=TCGA%20lower%20grade%20glioma%20and%20glioblastoma%20(GBMLGG)&amp;removeHub=https%3A%2F%2Fxena.treehouse.gi.ucsc.edu%3A443,GSE16011.

## Ethics Statement

The studies involving human participants were reviewed and approved by Ethics Committee of Xiangya Hospital. The patients/participants provided their written informed consent to participate in this study.

## Author Contributions

JS analyzed the data, performed computational coding, and wrote the manuscript. QM and WL involved in design of study, manuscript editing. HT performed the Q-PCR experiments and analyzed the results. CW, ML, XW, KX, YL, QX, CZ, and HL collected the data and samples. QL involved in design of study, manuscript review and editing, supervision of the entire work. All authors had approved the final manuscript.

### Conflict of Interest

The authors declare that the research was conducted in the absence of any commercial or financial relationships that could be construed as a potential conflict of interest.
