# Supplementary material for: LCTL Is a Prognostic Biomarker and Correlates With Stromal and Immune Infiltration in Gliomas
Source: Front Oncol. 2019 Oct 15;9:1083. doi: 10.3389/fonc.2019.01083 (PMC6803540; doi:10.3389/fonc.2019.01083)
Supplement: Supplementary file 1 [file Data_Sheet_1.zip › Supplementary tables and figure legends.docx]

Supplementary Material

# Supplementary Tables

**Supplementary Table S1.** The list of PCR primers.

**Supplementary Table S2.** Genetic alterations analysis of *LCTL* via cBioPortal in TCGA Merged Cohort of LGG and GBM.

**Supplementary Table S3.** Gene lists from the TCGA and GSE16011 data sets.

**Supplementary Table S4.** The results of Gene ontology (GO) analysis including BP, CC, and MF based on the TCGA data set.

**Supplementary Table S5.** The results of Gene ontology (GO) analysis including BP, CC, and MF based on the GSE16011 data set.

# Supplementary Figures

**Supplementary Figure S1.** *KL* and *KLB* expression in glioma and gene ontology (GO) enrichment of cellular component (CC) and molecular function (MF)

1. KL expression in lower grade glioma (LGG) and glioblastoma (GBM).
2. KLB expression in LGG and GBM.
3. Result of MF terms (the top 20 terms, p value < 0.01) analyzed by DAVID based on the TCGA dataset.
4. Result of MF terms (p value < 0.01) analyzed by DAVID based on the GSE16011 dataset.
5. Result of CC terms (the top 20 terms, p value < 0.01) analyzed by DAVID based on the TCGA dataset.
6. Result of CC terms (p value < 0.01) analyzed by DAVID based on the GSE16011 dataset. GO:0070062: extracellular exosome; GO:0031012: extracellular matrix; GO:0005925: focal adhesion; GO:0005788: endoplasmic reticulum lumen; GO:0005615: extracellular space; GO:0009897: external side of plasma membrane; GO:0009986: cell surface; GO:0016020: membrane; GO:0042470: melanosome; GO:0045121: membrane raft; GO:0031264: death-inducing signaling complex; GO:0005604: basement membrane; GO:0071556: integral component of lumenal side of endoplasmic reticulum membrane; GO:0005737: cytoplasm; GO:0005829: cytosol; GO:0042101: T cell receptor complex; GO:0042613: MHC class II protein complex; GO:0000139: Golgi membrane; GO:0005578: proteinaceous extracellular matrix; GO:0005783: endoplasmic reticulum; GO:0005913: cell-cell adherens junction; GO:0005515: protein binding; GO:0042803: protein homodimerization activity; GO:0002020: protease binding; GO:0005518: collagen binding; GO:0004197: cysteine-type endopeptidase activity; GO:0005178: integrin binding; GO:0003756: protein disulfide isomerase activity; GO:0001618: virus receptor activity; GO:0042802: identical protein binding; GO:0032395: MHC class II receptor activity; GO:0016641: oxidoreductase activity, acting on the CH-NH2 group of donors, oxygen as acceptor; GO:0043236: laminin binding; GO:0005123: death receptor binding; GO:0005509: calcium ion binding; GO:0001948: glycoprotein binding; GO:0001968: fibronectin binding; GO:0008201: heparin binding; GO:0005525: GTP binding; GO:0015026: coreceptor activity; GO:0048407: platelet-derived growth factor binding; GO:0004859: phospholipase inhibitor activity; GO:0098641: cadherin binding involved in cell-cell adhesion; GO:0030674: protein binding, bridging; GO:0019901: protein kinase binding; GO:0044548: S100 protein binding.

***P < 0.001, ns P > 0.05

**Supplementary Figure S2.** The heatmaps of *LCTL* related immune response and inflammatory response genes

1. Most immune response related genes were significantly positively correlated with *LCTL*.
2. Most inflammatory response related genes were significantly positively correlated with *LCTL*.

**Supplementary Figure S3.** The mRNA expression of *LCTL* significantly correlated with that of selected typical genes of immune and stromal scores in our 45 glioma samples (Pearson’s r, BH-adjust p value).

**Supplementary Figure S4.** Validation of the correlation between *LCTL* and immunosuppressive factors (selected 14 genes) in our 45 glioma samples (Pearson’s r, BH-adjust p value).

**Supplementary Figure S5.** Protein-protein interaction (PPI) analysis of *LCTL* and immune-related factors.

1. PPI analysis of *LCTL* via STRING
2. PPI analysis of *LCTL* and multiple factors (immunosuppressive cell recruitment, M2 differentiation, T cell exhaustion and epithelial-mesenchymal transition (EMT)) via STRING.
