# Supplementary figures and images for: LCTL Is a Prognostic Biomarker and Correlates With Stromal and Immune Infiltration in Gliomas
Source: Front Oncol. 2019 Oct 15;9:1083. doi: 10.3389/fonc.2019.01083 (PMC6803540; doi:10.3389/fonc.2019.01083)

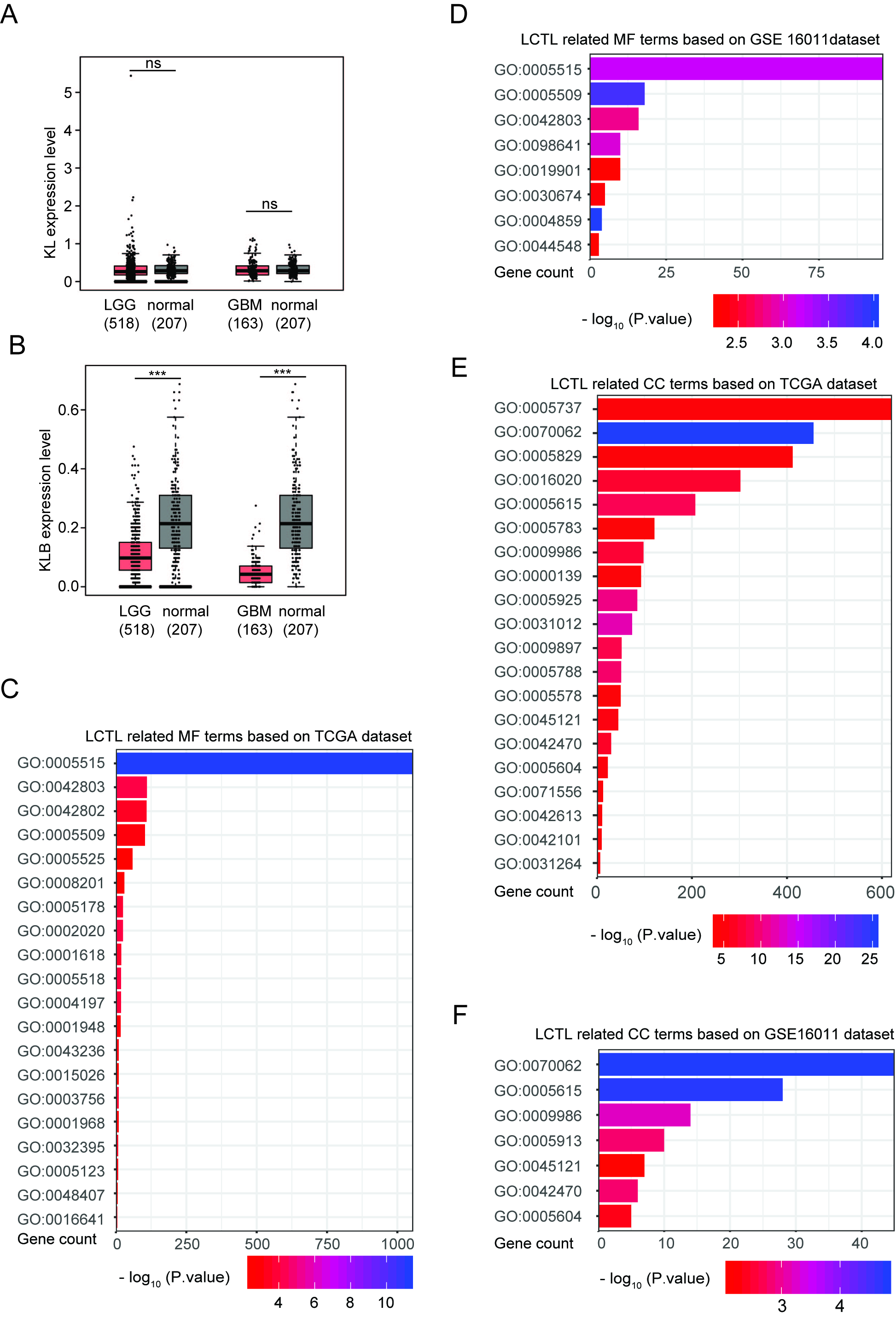

Supplement: Supplementary file 2 [file Image_1.tif]

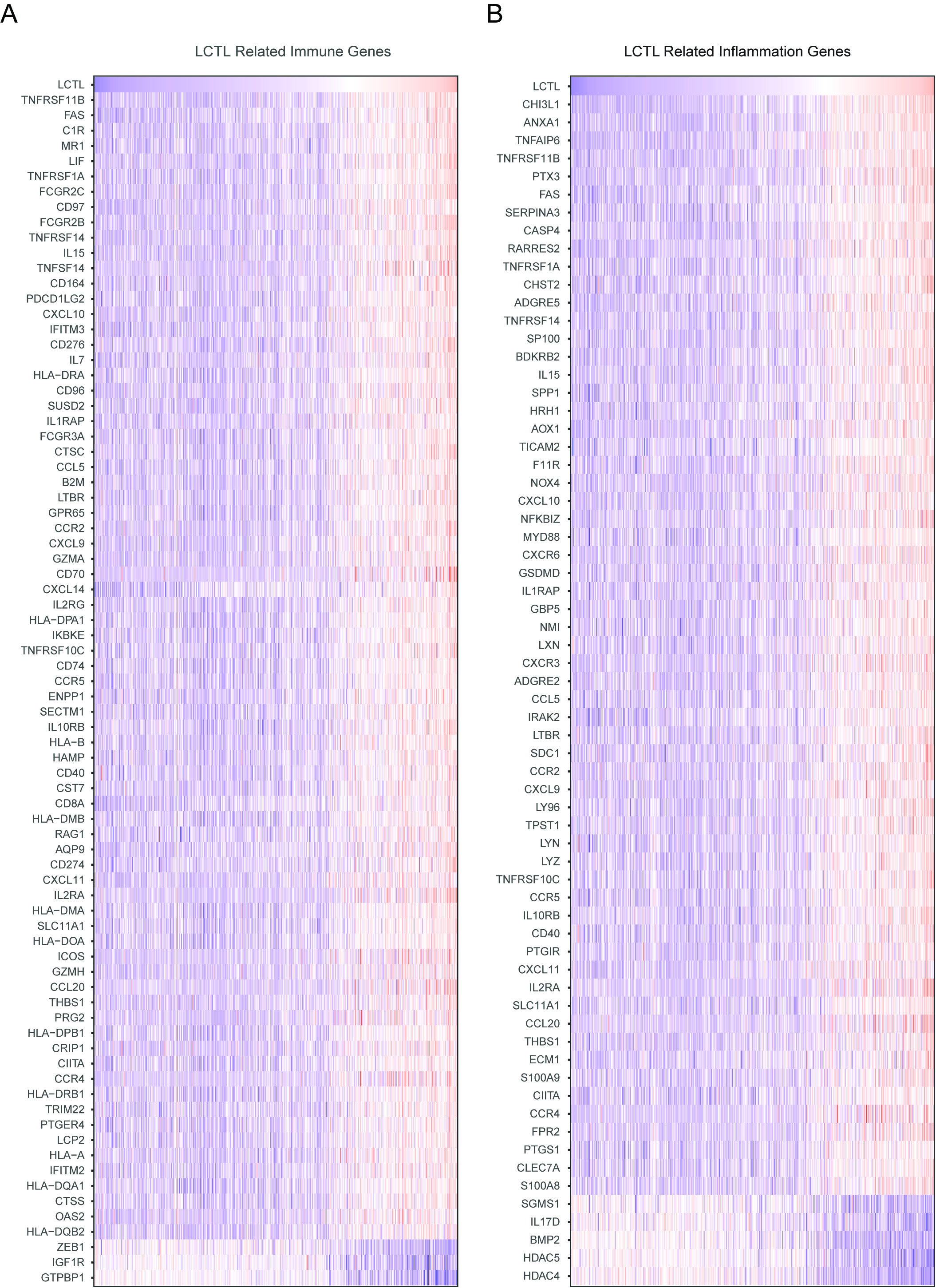

Supplement: Supplementary file 3 [file Image_2.JPEG]

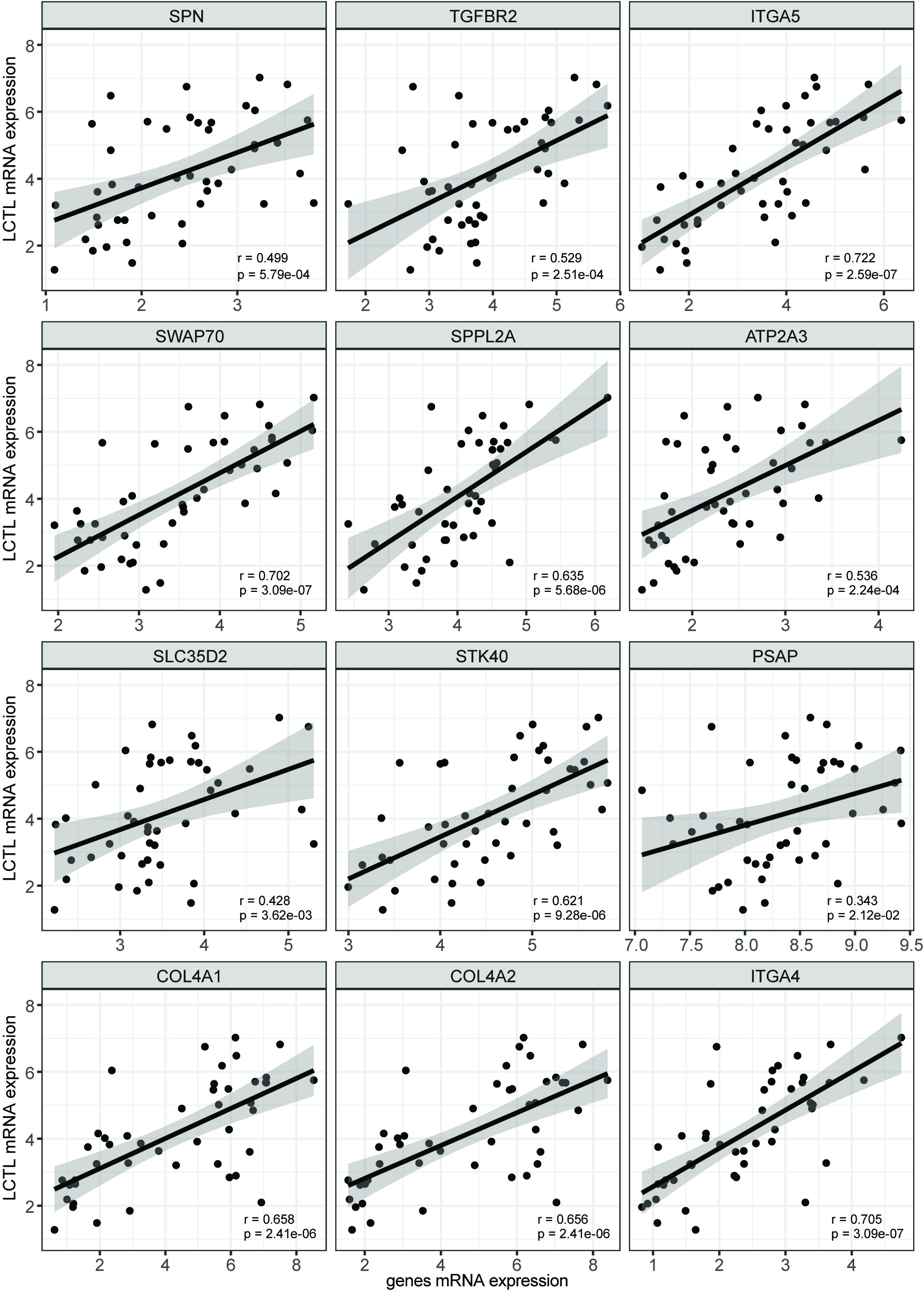

Supplement: Supplementary file 4 [file Image_3.JPEG]

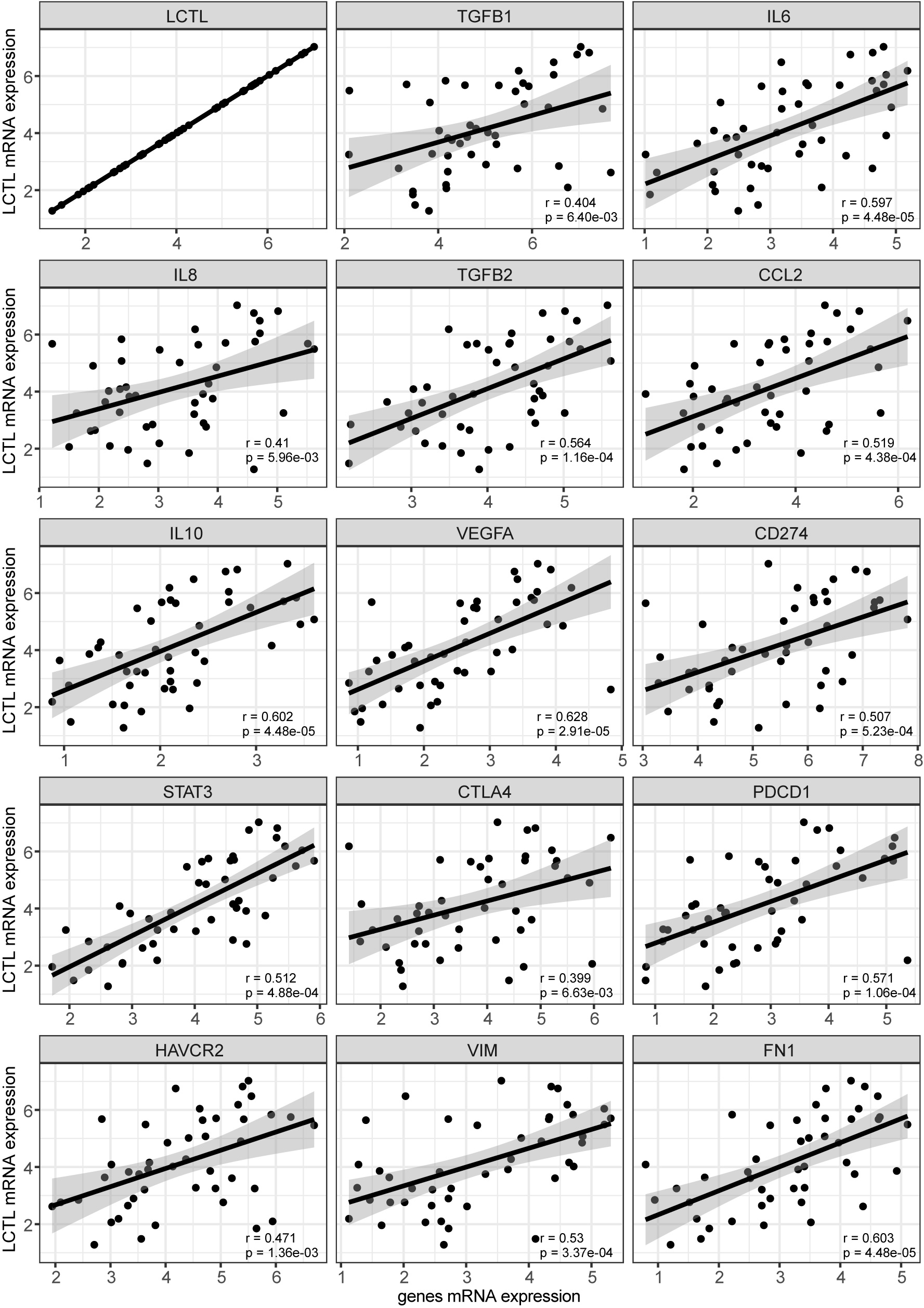

Supplement: Supplementary file 5 [file Image_4.JPEG]

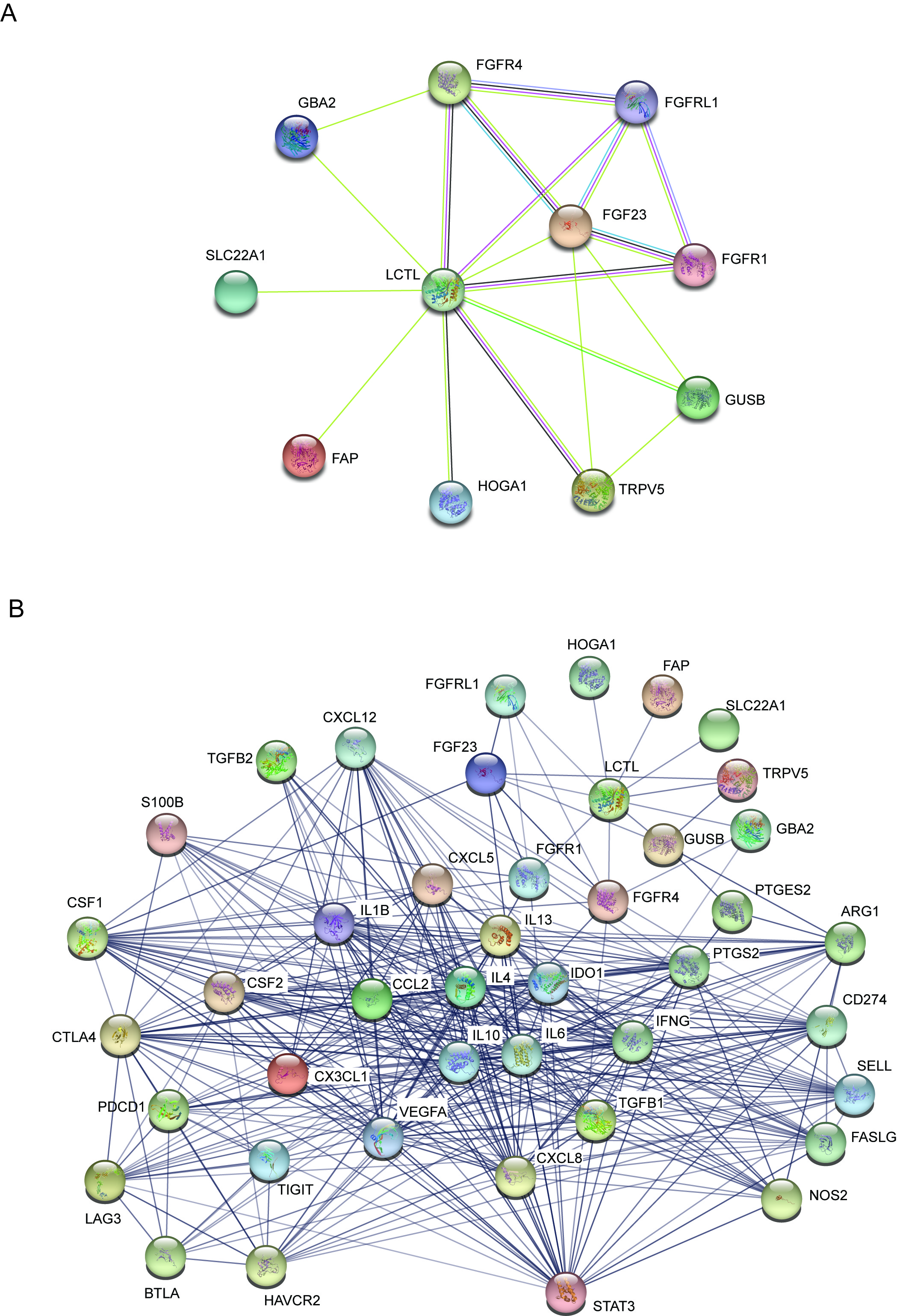

Supplement: Supplementary file 6 [file Image_5.JPEG]
